# Citizen science approaches in the development of post-stroke physical activity interventions: A scoping review

**DOI:** 10.1371/journal.pone.0329948

**Published:** 2025-08-20

**Authors:** Martijn Bakker, Rhoda Schuling, Rienk Dekker, Leonie A. Krops, Johan de Jong

**Affiliations:** 1 Department of Rehabilitation Medicine, University Medical Center Groningen, University of Groningen, Groningen, The Netherlands; 2 School of Sport Studies, Hanze University of Applied Sciences, Groningen, The Netherlands; 3 Department of Human Movement Sciences, University Medical Center Groningen, University of Groningen, Groningen, The Netherlands; La Trobe University - Melbourne Campus: La Trobe University, AUSTRALIA

## Abstract

**Background:**

Stroke is a major cause of disability globally, with high recurrence rates despite the implementation of secondary prevention strategies. Promoting physical activity and reducing sedentary behaviour are critical to mitigate these risks. Collaborative research approaches, including citizen science, offer promising methods for developing more effective and sustainable interventions by leveraging patient insights and lived experiences across different research stages.

**Objectives:**

This scoping review explored the application of citizen science approaches in developing interventions targeting physical activity and sedentary behaviour for people with stroke.

**Methods:**

Following Arksey and O’Malley’s framework and the PRISMA-ScR checklist, five databases were searched. We included empirical studies involving stroke patients in research on physical activity or sedentary behaviour interventions. Data was extracted on terminology, collaboration methods, and participant roles and analysed using the Participation Matrix framework. Methodological rigor was assessed using the CASP qualitative checklist.

**Results:**

Fourteen studies were included, most published after 2020 and originating from diverse countries. Terms like “co-design,” “co-creation,” and “patient and public involvement” were prevalent, but “citizen science” was not explicitly mentioned. Methods for active involvement of stroke patients included focus groups, workshops, and advisory panels. Stroke patients primarily participated as advisors or partners during intervention design, with minimal involvement in early research stages, data analysis, or dissemination. Researchers predominantly held decision-making roles.

**Conclusions:**

Citizen science in stroke research is still developing, with limited patient involvement across research phases. Expanding the depth and scope of patient involvement could enhance the relevance and long-term impact of interventions.

## Introduction

Stroke is a leading global cause of disability, significantly impacting individuals’ physical and functional abilities and reducing quality of life [1,2]. Multiple modifiable risk factors such as physical inactivity, sedentariness, smoking, poor diet, and alcohol intake are known to contribute to stroke incidence [3]. Moreover, the recurrence rate in stroke is high, reaching up to 50%, which can compound physical and cognitive disabilities while increasing healthcare costs [4]. To address these issues, effective secondary prevention strategies are essential to reduce the risk of stroke recurrence and improve long-term functional outcomes [5,6]. Among secondary prevention strategies, promoting physical activity and reducing sedentary behaviour are crucial measures to reduce the risk of stroke recurrence and increase their overall health and quality of life [7,8].

Implementing effective interventions for stroke survivors to adopt and sustain active behaviour presents a major challenge, especially after transitioning from a rehabilitation setting to peoples’ living environment [9,10]. Despite the increase of secondary prevention strategies, stroke recurrence rates have remained unchanged over the last 20 years [11]. Behavioural change complexities emphasize the necessity for interventions that seamlessly integrate into patients’ daily lives and routines in order to enhance long-term maintenance. Collaborative approaches that engage healthcare professionals, policymakers, researchers, and especially patients are gaining traction for developing evidence-based interventions [12–16]. This shift from research conducted “for” patients to research conducted “with” patients highlights the value of patients’ lived experiences and insights. By involving patients directly, these approaches seek to make interventions more impactful and better suited to integration into daily life, thus addressing the specific behavioural challenges in secondary stroke prevention.

Citizen science approaches are promising for actively involving patients in research. Despite the apparent lack of uniformity in the definition of citizen science (14), most sources agree that citizen science is about involving citizens in science. This aligns with the first of 10 key principles set by the European Citizen Science Association (ECSA): *‘’Citizen science projects actively involve citizens in scientific endeavour that generates new knowledge or understanding. Citizens may act as contributors, collaborators, or as project leader and have a meaningful role in the project”* [17]. Throughout this current study, citizen science is defined and considered as an umbrella term encompassing a range of participatory models and activities where individuals who are not professional researchers actively participate in and contribute to various stages of the research process [17–19]. The collaboration between citizens and scientists (and other stakeholders) can have many forms, allowing citizen science frameworks to appear in diverse methodologies. Multiple authors have outlined and categorized the range of approaches that could be classified as citizen science, varying along a continuum of intensity in involvement or collaboration. This continuum ranges from crowdsourcing, where citizens solely contribute to data collection, to more intensive forms where citizens co-create or even lead the research process [20,21].

Although citizen science approaches have their roots in natural sciences (e.g., ecology, environmental sciences), the term ‘’citizen science” has become more common in the health domain over the past decade [22]. For example, the *Our Voice* initiative has involved community members to drive changes in local environments to support physical activity [23]. Another example is the *Healthy Slotermeer* project in the Netherlands, where local residents were trained to interview fellow residents [24]. It is part of a paradigm shift of more active involvement of participants in research and builds upon a tradition of patient participation in health research. Participatory methodologies in medical and health research, such as Patient and Public Involvement (PPI) and Community-Based Participatory Research (CBPR), will for the purposes of this review be considered forms of citizen science [25–27]. These methodologies share a common emphasis on engaging participants in the research process, with PPI focusing on patients’ experiences and CBPR leveraging the expertise of community members in public health projects.

In recognition of the value of the expertise and lived experiences of patients, many funding agencies now require patient participation in science. In secondary stroke prevention, citizen science approaches may enhance strategies by fostering a sense of ownership and knowledge of patients and increasing the applicability and therefore sustainable adoption of these strategies in daily life situations. However, no single approach has emerged as the standard from the many different ways to conduct citizen science, and many questions remain about the methods used and the results they produce.

Considering the increased focus on citizen science approaches in health research, as well as the diverse ways in which such initiatives are developing, the current scoping review was conducted to explore this field. Although previous scoping reviews have explored participatory approaches in stroke research, such as co-design practices [28] and stroke patient and stakeholder engagement models [29], they did not examine the specific roles of patients and researchers throughout the research process. They also did not assess the methods used to involve patients. Importantly, neither study considered the full spectrum of citizen science approaches or classified the intensity and breadth of involvement. This highlights a significant knowledge gap regarding how citizen science approaches are operationalized and conceptualized within stroke intervention research.

We aimed to provide a comprehensive overview of citizen science approaches and elements in the context of physical activity and/or sedentary behaviour interventions for stroke patients. Specifically, we were interested to see at what end of the citizen science continuum the current research activities manifest, given the promise that intensive collaboration with patients would lead to relevant and acceptable interventions that better fit into patients’ daily lives and routines [30]. The overarching question of this review is: *How have citizen science approaches been applied within stroke research focusing on intervention development targeting physical activity and sedentary behaviour?* The sub-questions are:

I What terminology is used to define citizen science approaches within stroke research?
II Which methods are used to achieve active involvement of stroke patients?
III What was the role of stroke patients in the research process?

## Materials and methods

We designed and conducted this scoping review according to the recommendations by Arksey and O’Malley [31] and Levac et al. [32] and followed the Preferred Reporting Items for Systematic Reviews and Meta-Analyses-Extension for Scoping Reviews (PRISMA-ScR) for reporting (S1 File*)* [33]. We considered a scoping review the most suitable method, as it allows for a broad and systematic exploration of the existing literature, enabling the identification of various forms of citizen science approaches and their applications in stroke research. The following five phases were included in this review: i) identifying the research question, ii) identifying relevant studies, iii) study selection, iv) data collection, and v) collating, summarizing, and reporting the results*.* The objectives, inclusion criteria and methods for this scoping review were specified a-priori and documented in a protocol, shared at Open Science Framework (https://osf.io/guha4/).

### Identifying relevant studies

The development of the search strategy was led by MB with the support of an information specialist of the University library (Hanze University of Applied Sciences). A comprehensive list of search terms was identified through searches on PubMed using medical subject headings (MeSH) and keywords. Different combinations of search terms were tested and discussed during regular meetings with the research team. The final version of the search strategy consisted of a combination of the following three concepts: stroke, physical (in)activity and citizen science. We made the decision to include studies which used participatory approaches akin to citizen science, also when they did not explicitly used the term ‘’citizen science.” The ECSA principles were used to determine if the methods could be classified as a citizen science approach [17]. This aligns with ongoing debates surrounding the definition of citizen science and the recognition that diverse terms and methodologies are often used within this framework [19,34,35]. Truncation and proximity operators were employed to increase the sensitivity of the search (see S2 File for the exact search strategy). The databases PubMed, Embase, CINAHL, PsycINFO and Scopus were systematically searched until the 21^st^ of March 2024. Additionally, reference lists of included articles were screened to identify additional studies. In March 2025, an updated search was performed to ensure comprehensiveness. Articles were included if they met the following inclusion criteria: i) empirical studies, ii) a clear description of the citizen science approach, iii) focus on physical activity or sedentary behaviour of stroke patients, iv) design of an intervention. Articles were excluded based on the following criteria: i) absence of actual involvement of stroke patients in the research process, ii) conference abstracts and protocols. There were no further restrictions on study design or language of the article. However, during the screening process, no full-text articles written in languages other than English were retrieved

### Study selection

**Fig 1:**
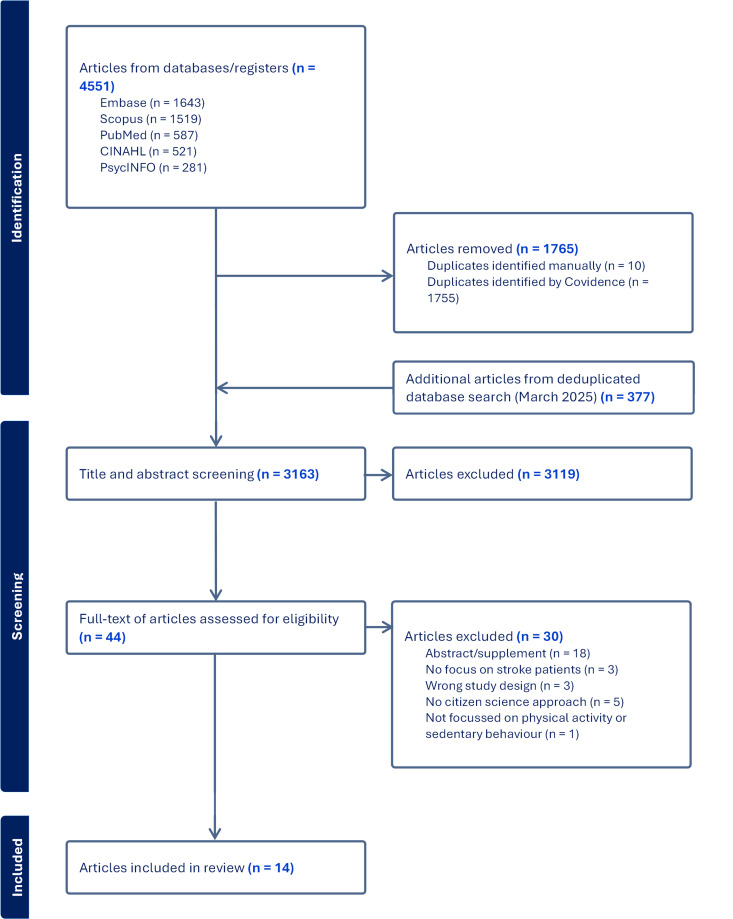
Flowchart of screening and review process of included articles. n = number of articles.

All citations were imported into Covidence® for de-duplication and screening [36]. An initial pilot test of ten references was conducted to ensure clarity and consistency in the application of the inclusion and exclusion criteria during the title and abstract screening. Uncertainties were discussed and the criteria were refined through discussion. All titles and abstracts were screened by two independent reviewers (MB and RD or JJ) and any disagreements between the reviewers were discussed until consensus was reached. Eligibility of full text articles were screened by two researchers (MB & RS) independently and disagreements were discussed. In case of doubt, the articles were also discussed with the whole research team. Inter-rater reliability between reviewers was calculated and showed substantial agreement: κ = 0.68 for title/abstract screening and κ = 0.77 for full-text screening. Fig 1 shows the flow of the study selection process.

### Critical appraisal

A critical appraisal of qualitative studies was performed using the Critical Appraisal Skills Programme (CASP) checklist for qualitative studies to assess the quality of studies [37]. Although quality assessment is not mandatory in scoping reviews [38], we did include it to better understand the strengths and limitations of the included articles. This decision was made in light of the substantial variability in citizen science methodologies and the absence of a universally accepted gold standard for participatory research. We used the results of the critical appraisal to contextualize our findings rather than exclude studies based on their quality scores. The CASP tool was applied by two independent reviewers (MB and LK) during data extraction. MB screened all included articles, and LK independently screened 5 articles and checked the remaining 8 articles, after which inconsistencies were discussed.

### Data collection

The first author (MB) extracted the data using a data extraction template which was developed in Covidence® (S3 File). All extracted data was reviewed and complemented by a second researcher (LK). Data was extracted on study characteristics (author, year of publication, country of origin), demographics of citizen scientists (age, gender, severity of stroke), methods (terminology, recruitment, setting, methods of collaboration), the role of stroke patients in the research process and the intensity of participation.

### Collating, summarizing, and reporting the results

Data synthesis was performed by the first author (MB) and refined through regular discussions with the research team. General characteristics of the included studies were descriptively analysed. We also descriptively analysed the aims and methods of the included studies to address the first and second sub-question. To answer the third sub-question, we utilized and adapted the *Participation Matrix* developed by Smits et al. to perform a narrative analysis [39]. This model defines five distinct roles that patients and researchers can occupy in research: *Listener, Co-thinker, Advisor, Partner,* and *Decision-maker*. We added a sixth role: *Non-involvement*. This role was added to account for phases of research where patients may have no active engagement beyond being subjects in the study, such as participation in a randomised controlled trial (RCT) without further involvement in the research process. Each role reflects varying degrees of engagement, from no involvement (*Non-involvement*) to leading the research process (*Decision-maker*), in line with the principles of citizen science.

To evaluate their role, we assessed how stroke patients were integrated as citizen scientists across three key research phases: i) *Preparation phase* (including formulating the research question and the set-up of the study design), ii) *Execution phase* (including data collection and data analysis), and iii) *Implementation and reporting phase* (including reporting results and dissemination of results). Each study was systematically evaluated to assign the appropriate role based on the nature of the contributions made by stroke patients. This approach allowed us to systematically assess and map the role of stroke patients across different research phases. By adopting this categorization, we aimed to gain deeper insights into their specific roles in shaping interventions focused on physical activity or sedentary behaviour.

## Results

The final search retrieved 4928 studies and after deduplication 3163 studies were eligible for title and abstract screening. Title and abstract screening were conducted using the eligibility criteria, resulting in the selection of 44 full-text articles. Following the full-text screening, 14 articles were included in this review (Fig 1). A total of 30 full-text articles were excluded at this stage. Reasons for exclusion included: article types such as conference abstracts or study protocols; lack of actual engagement of stroke patients in the research process (e.g., patients only acted as subjects); or no focus on physical activity or sedentary behaviour. A summary of the characteristics of the included studies is presented in Table 1.

**Table 1:** Summary of study characteristics.

Authors	Country	Design	Terminology	Focus of study	Setting of the study	Involved stroke patients (n)	Other stakeholders	Methods of collaboration
Bodilsen et al. 2023 [40]	Denmark	Qualitative	Co-creation	Develop an intervention to reduce SB and increase PA	Rehabilitation	3	Relatives, physiotherapists, occupational therapists, nurses	Workshop and focus group
Driver et al. 2020 [41]	USA	Qualitative	Community-Based Participatory Research approach	Modify a Healthy lifestyle intervention (PA is part of the intervention)	Community	6	Care partner, physiatrists, rehabilitation therapists, neuropsychologist, exercise specialist, dieticians, health and wellness practitioner and representatives	Small subgroup meetings; advisory board
Ezeugwu & Manns 2020 [42]	Canada	Qualitative	Not specified	Develop an intervention to reduce SB	Community	13	Expert stroke clinicians	Interviews; feedback sessions; stakeholder meetings
Hall et al. 2020 [43]	UK	Qualitative	Co-production	Develop an intervention to reduce SB	Starting in an inpatient setting and following through discharge into the community	14	Staff members of the stroke services, researchers, caregivers, inpatient and community stroke service staff and exercise instructors.	Co-production workshops
Heron et al. 2021 [44]	UK	Qualitative	Person-based approach	Develop a digital lifestyle modification intervention (PA is part of the program)	Community	32	–	Focus groups; think aloud interviews
Irvine et al. 2023 [45]	UK	Qualitative	Co-design and Patient and Public involvement	Development of a text message intervention to promote PA and exercise.	Community	8	Therapists, academics and representatives of stroke groups	Collaborative working Group, group meetings, surveys, interviews
Kwah et al. 2024 [46]	Singapore	Qualitative	Co-design	Development of a complex intervention targeted at improving PA after stroke	Community	13	Caregivers, physiotherapists, neuropsychologist, User Experience (UX) designer	Face-to-face survey, interviews and co-design workshops
Levy et al. 2022 [47]	Australia	Mixed-methods (development part is qualitative)	Proctor’s framework for implementation research	Develop and evaluate an exercise-based group for stroke survivors and their carers	Inpatient	30	Carers of stroke survivors, physiotherapists, occupational therapists, allied health assistants and a nurse	Interviews, focus group, surveys and meetings
Lund et al. 2012 [48]	Norway	Qualitative	Person-centred process	Develop a lifestyle intervention (PA is one of the identified themes)	Community	132	–	Interviews and group sessions
Moore et al. 2022 [49]	UK	Qualitative	Engagement, consultation, co-design and person-centred	Develop an intervention targeting PA and SB	Community	21	Physiotherapists, technical instructors and physiotherapy assistants	Focus groups, consultation workshops, patient and carer panel for feedback
Morris et al. 2022 [50]	UK	Qualitative	Person-centred design and Patient and Public Involvement	Develop an intervention to increase PA	Community	23	Companions of stroke patients, physiotherapists, occupational therapists, stroke nurses, and local exercise services coordinators	Focus group, stakeholder and expert consultation
Olafsdottir et al. 2020 [51]	Iceland, Sweden and Finland	Qualitative	Human-centred design and co-design	Develop an intervention to promote home-based exercise and PA	Community	7	Informal caregivers, rehabilitation professionals	Interviews, focus group
Ramage et al. 2022 [52]	Australia	Qualitative	Co-production	Develop a telehealth exercise intervention (PA)	Community	11	Caregivers, healthcare workers, behaviour change researcher	Workshops, interviews and collaborative decision making
Sakakibara et al. 2017 [53]	Canada	Qualitative	Stakeholder review and revision, patient-centred	Healthy lifestyle (PA is part of the program)	Community	Not specified	Stroke patient groups, advocacy groups, health professionals, and other researchers	Feedback session

Abbreviations: PA = physical activity; SB = sedentary behaviour.

### Study characteristics

The majority of the articles (n = 12) were published after 2020, with five conducted in the United Kingdom, followed by contributions from Canada (n = 2), Australia (n = 2), Denmark (n = 1), the USA (n = 1), Norway (n = 1), Iceland (n = 1) and Singapore (n = 1). Of the 14 included articles, 13 employed a qualitative research design, while one study utilized a mixed-methods approach. All studies focused on developing interventions to increase physical activity or reduce sedentary behaviour. While 11 studies targeting community or home-based settings three studies developed an intervention targeting stroke patients in an inpatient setting. Sample sizes varied substantially, ranging from 3 to 132 stroke patients. Two studies only involved stroke patients, the other 12 studies also involved different stakeholders, e.g.,: caregivers, healthcare professionals, advocacy groups, and exercise instructors in the development process of the intervention.

### Critical appraisal

Using the CASP tool, a total of 13 studies, excluding one mixed-methods study [47], were appraised to assess their methodological rigor. Most studies demonstrated strengths, particularly in articulating clear research objectives, employing appropriate study designs, and addressing ethical considerations, which are critical for ensuring transparency and rigor in qualitative research. Ten out of 13 studies lacked a clear discussion of the researcher-participant relationship and did not provide sufficient detail regarding the rigor of their data analysis processes (Table 2).

**Table 2:** Overview of critical appraisal (CASP) of included studies.

	Clear aims statement	Appropriate methodology	Appropriate research design	Appropriate recruitment	Appropriate data collection	Researcher-participant relationship considered	Ethical issues considered	Rigorous data analysis	Cleare statement of findings
Bodilsen et al. 2023 [40]	Y	Y	Y	Y	Y	N	Y	Y	Y
Driver et al. 2020 [41]	Y	Y	N	N	N	N	?	N	N
Ezeugwu & Manns 2020 [42]	Y	Y	Y	Y	Y	N	Y	?	Y
Hall et al. 2020 [43]	Y	Y	Y	Y	Y	Y	Y	Y	Y
Heron et al. 2021 [44]	Y	Y	Y	Y	Y	?	Y	N	Y
Irvine et al. 2023 [45]	Y	Y	Y	Y	?	?	Y	?	Y
Kwah et al. 2024 [46]	Y	Y	Y	Y	Y	?	?	Y	Y
Lund et al. 2012 [48]	Y	Y	Y	Y	?	Y	Y	?	Y
Moore et al. 2022 [49]	Y	Y	Y	Y	Y	?	Y	?	Y
Morris et al. 2022 [50]	Y	Y	Y	Y	Y	?	Y	Y	Y
Olafsdottir et al. 2020 [51]	Y	Y	Y	Y	Y	?	Y	?	Y
Ramage et al. 2022 [52]	Y	Y	Y	?	Y	Y	Y	?	Y
Sakakibara et al. 2017 [53]	Y	Y	Y	?	?	?	Y	?	Y

Abbreviations: Y=yes; N=no;?=can’t tell.

### Terminology

The included articles used a range of different terms to describe the participatory character of the study, namely co-design, person-centred design, patient and public involvement, co-creation, co-production, community-based participatory research approach, person-based approach, human-centred design and stakeholder review. The term citizen science was not mentioned in any of the included articles.

### Collaborative methods

The included studies demonstrated a variation of methods to actively involve stroke patients in the development of interventions. Common methods included interviews (n = 8), focus groups (n = 6), workshops (n = 5) (e.g., co-production workshops in [43] and [52]), and feedback sessions (n = 3). Other approaches, such as the use of patient and carer panels, an advisory board and a collaborative working group highlighted efforts to tailor engagement methods to participant needs. The inclusion of diverse stakeholders, such as caregivers and healthcare professionals, complemented patient involvement and ensured a multidisciplinary perspective.

For instance, Hall et al. [43] conducted a series of co-production workshops involving stroke patients, caregivers, and clinicians to co-develop an intervention aimed at reducing sedentary behaviour. In contrast, Irvine et al. [45] utilized the expertise of a collaborative working group (CWG), comprising of individuals with stroke, rehabilitation therapists, and academics, and a structured review process to co-design a text message-based physical activity intervention.

### Partnership roles

**Fig 2:**
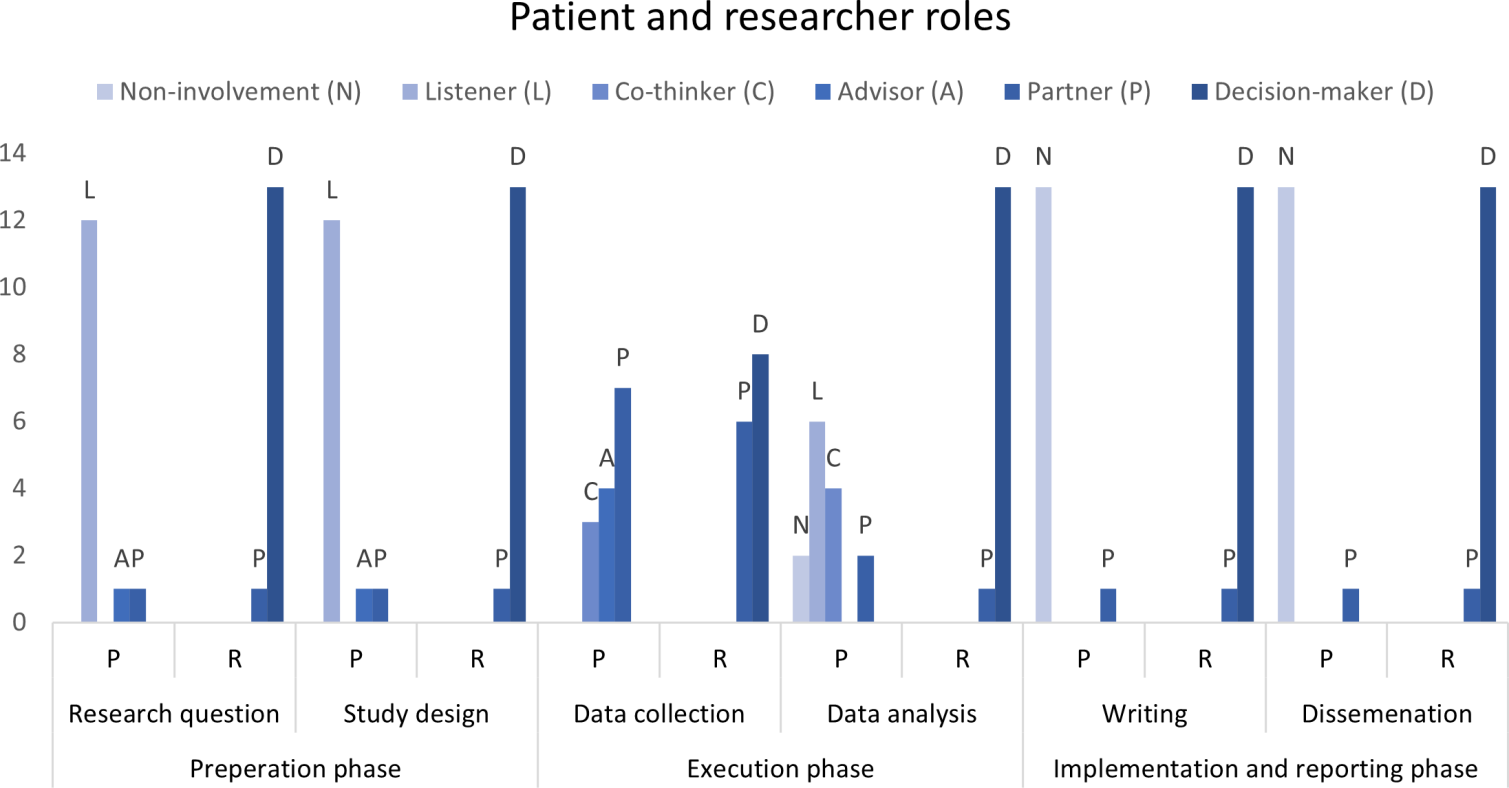
Roles of stroke patients and researcher throughout the research phases. The colour gradient, from light to dark, indicates an increasing level of involvement in the research process.

The roles of stroke patients and researchers were systematically categorized using the adapted Participation Matrix developed by Smits et al. [39] (see Fig 2 and S4 File). The role of stroke patients differed throughout the three research phases. In the preparation phase, stroke patients were most frequently assigned the role of Listener (n = 12), reflecting their limited engagement in this phase. A minority of studies (n = 2) demonstrated more active involvement of patients, such as advisor or co-thinker, particularly when patients contributed perspectives on refining research questions or intervention objectives.

In the execution phase, which included data collection and analysis, the role of stroke patients shifted towards more active involvement. Patients most commonly acted as advisors (n = 4) or partners (n = 7). Their contributions were especially notable in the context of data collection, where patients played a central role, such as being active in workshops, focus groups, or community-based assessments. However, their involvement in data analysis was less frequent and primarily limited to studies that explicitly integrated participatory methodologies.

The implementation and reporting phase displayed the least consistent involvement of stroke patients. One study reported a stroke patient serving as a co-author, actively contributing to writing and dissemination, which we categorized as the role of partner. However, in most cases, patients were either absent or minimally involved during this phase.

Conversely, the roles of researchers were predominantly stable across all phases of the research process. Researchers were consistently assigned the role of decision-maker, reflecting their leadership in steering the research process and making final decisions.

## Discussion

This review sought to examine the extent and nature of citizen science approaches in the development of interventions targeting physical activity and/or sedentary behaviour for stroke patients. We specifically focussed on the terminology used to describe the participatory approach of the study, the methods used for engaging stroke patients and the roles of stroke patients and researchers during the research process. We identified 14 articles with methods that align with the concept of citizen science. The majority of the included studies were published after 2020 and conducted in the United Kingdom and other western countries. This rise of citizen science approaches in post stroke physical activity interventions may reflect increased policy support and funding, such as initiatives led by organizations as INVOLVE in the UK [54], which promote active citizen involvement in research processes to enhance relevance and inclusivity. Our findings indicate that while diverse methods and terminologies are used, there remain significant gaps in the depth of engagement and the stages of the research process in which stroke patients are involved.

The included articles showed a wide variation in terminology used to describe the citizen science approaches, underscoring the lack of standardization in this field. Similar inconsistencies in terminology have also been reported in other review studies examining participatory approaches in stroke research [28,29,55]. Interestingly, the term “citizen science” was not used in any of the included studies, despite its rise in health research, and for example in chronic disease prevention studies [22,56]. This absence may reflect a lack of familiarity with the concept of citizen science among stroke researchers or an association with research practices distinct from terms like “patient involvement” or “public participation”. Introducing the term ‘’citizen science” into stroke research may present an opportunity to broaden the scope of the current participatory approaches, encouraging more active collaboration between researchers and patients. By addressing gaps in terminology and raising awareness of frameworks like citizen science, the field could move towards greater clarity and consistency, enabling better cross-study comparisons and improving patient-centred outcomes in stroke research.

In addition to the variation in terminology, the studies included in this review used diverse methods to engage stroke patients, such as workshops, interviews, focus groups, and advisory panels. While these methods provide valuable insights into stroke patients’ experiences and perspectives, they often limit patients to advisory and consultative roles. These methods, while beneficial in gathering patient input, tend to fall short of fostering more collaborative and equal partnerships. More creative and visual methodologies, e.g., storytelling, may help achieve more effective engagement of stroke patients and empower them as active contributors [57,58]. Such agency over their rehabilitation process may prove useful when developing secondary prevention strategies that more accurately fit patients’ needs.

Building on this need for more inclusive engagement, our analysis of partnership roles across the different research phases revealed a substantial difference in the degree of involvement between stroke patients and researchers. We observed that the role of researchers was constant: they were predominantly in the role of decision-making. Stroke patients were primarily engaged during the execution phase, often as advisors or partners, contributing to data collection and providing feedback on concepts of interventions designed by the research team. Achieving meaningful collaboration between researchers and stroke patients seems to remain a considerable challenge, as seems to be a tendency for citizen science in general [59]. However, the study of Ramage et al. [52] provided an exception, wherein a stroke patient was not only a genuine partner but also co-author of the publication. In this instance, the roles and dynamics between researchers and stroke patient were more evenly distributed, illustrating a different model of collaboration.

Overall, there was little to no involvement of stroke patients in the preparation phase, where study design decisions are made, and the implementation and dissemination phases, which are essential for translating research into practice. This pattern raises concerns, as active involvement in these critical phases can ensure that research addresses the most relevant questions and that interventions are effectively integrated and sustained into real-world settings [60,61]. The methods used to engage patients directly influence their roles in the research process; if research teams are committed to fostering deeper collaboration, they need to carefully considered and define these roles early on, before the study begins. The purpose and format of the collaboration should be clear to both patients and researchers [62]. This ensures that collaboration is built into the structure of the study, guiding the design and implementation of research in a way that promotes engagement. Additionally, the researcher-participant dynamics should be reported in detail.

A key challenge in more intensive partnership roles is moving beyond traditional hierarchical relationships between researchers and patients. These power dynamics between patients as partners and the research team are also recognized in the review of Bird et al. [63]. Especially within the health sector, which is traditionally structured hierarchically, establishing reciprocity and fostering strong relationships between researchers and patients is vital for the success of participatory research designs [64,65]. True engagement requires researchers to adopt a mindset characterized by openness, adaptability, and sensitivity to the lived experiences of stroke survivors [66]. This shift involves recognizing that patients are not merely informants but can be active collaborators whose insights could improve the research process and its outcomes. However, the feasibility of such engagement is not without challenges. Stroke survivors may experience cognitive impairments, aphasia or fatigue, which can limit their capacity to participate in a meaningful way. These barriers, while acknowledged in stroke rehabilitation more broadly, are often underexplored in participatory research. The studies in our review provided limited insights into how such constraints were accommodated, suggesting a need for more adaptive and inclusive methodological strategies. Importantly, the utility of citizen science approaches lies not in a ‘one-size-fits-all’ model but in its adaptability.

Finally, few studies evaluated the impact of the participatory methods used. Viewed through the lens of the ECSA principles, several key elements of citizen science were underrepresented. Participant involvement was often limited to specific phases such as consultation or feedback (principle 4), with little evidence of engagement in agenda-setting or dissemination. Feedback to participants (principle 5) and formal acknowledgment in publications (principle 8) were inconsistently reported. Moreover, mutual benefit for both researchers and participants (principle 3) was rarely described and only a minority of studies evaluated participant experience or broader impact (principle 9). These gaps suggest that while participatory methods were used, few studies fully embraced the broader potential of citizen science approaches. Systematically evaluating the effectiveness of participatory approaches, both in terms of process and outcomes, could help researchers make more informed, context-sensitive methodological choices, and enhance the design and impact of future interventions.

### Strengths and limitations

This review provides an overview of how citizen science approaches have been applied in stroke research focussing on intervention development to enhance physical activity or reduce sedentary behaviour. By analysing existing studies, we highlight both the progress made and the extent to which these approaches have been integrated. Understanding this landscape can help researchers decide whether and how to implement citizen science in their own studies. Another strength of this review is the structured analysis of partnership roles using the Participation Matrix. This framework allowed us to make a systematic categorization of patient and researcher roles in research. However, the categorization was sometimes challenging due to insufficient available detail on patient roles across research stages. This underscores the need for clearer reporting on roles, responsibilities, and decision-making processes to enhance transparency and accountability in participatory research.

A limitation may be that selection bias may have influenced the findings, as we included studies that aligned with participatory principles of citizen science even if they did not explicitly used the term ‘citizen science.’ Despite using a broad range of search terms related to participatory research, some relevant studies may have been missed due to the varied terminology in this field. Our approach is in line with ongoing discussions and debates about the definition of citizen science. It broadens the scope of the review but may also introduce heterogeneity in the included methodologies. Additionally, challenges such as cognitive impairments and accessibility barriers may have influenced the depth of involvement of patients reported in the included studies. Notably, the included studies provided limited insights into these barriers, indicating a gap in understanding how participatory methods can be adapted to accommodate stroke-related disabilities. Addressing these challenges requires intentional strategies to support patient engagement at all research stages.

## Conclusion

While this review demonstrates that citizen science approaches are becoming increasingly prevalent in stroke research, it also reveals significant gaps in the depth and quality of involvement of stroke patients as partners in research. Although patients often contribute valuable input, their roles are typically confined to advisory or consultative levels, with limited engagement in later phases of research, or in decision-making roles. Future research should explore how participatory approaches can be adapted to be more inclusive of this population. Moving forward, it is crucial for stroke researchers to adopt more inclusive models of citizen science that allow for deeper collaboration and shared decision-making across all phases of the research process. Future research should establish clearer frameworks for participatory roles and incorporate structured evaluation of the effectiveness. By doing so, we can ensure that interventions not only reflect the perspectives and needs of stroke patients but are also more likely to be adopted and sustained in real-world settings, ultimately improving long-term outcomes for stroke survivors.

## Supporting information

S1 File PRISMA-ScR checklist. (DOCX)

S2 File Search strategy different databases. (DOCX)

S3 File Template data extraction. (DOCX)

S4 File Analysis of partnership roles. (DOCX)
