# Supplementary material for: Citizen science approaches in the development of post-stroke physical activity interventions: A scoping review
Source: PLoS One. 2025 Aug 20;20(8):e0329948. doi: 10.1371/journal.pone.0329948 (PMC12367154; doi:10.1371/journal.pone.0329948)
Supplement: S2 File — (DOCX) [file pone.0329948.s002.docx]

**Pubmed**
21-03-2024: 587 results
06-03-2025: 635 results

Stroke[Mesh] OR poststroke[tiab] OR CVA[tiab] OR "cerebrovascular accident*"[tiab] OR stroke[tiab]

AND

"Citizen Science"[Mesh] OR "Community-Based Participatory Research"[Mesh] OR "Patient Participation"[Mesh] OR "Stakeholder Participation"[Mesh] OR "Patient Involvement"[tiab:~3] OR "patient and public involvement"[tiab:~3] OR "Patient engagement"[tiab:~3] OR "patient partnership"[tiab:~3] OR "Stakeholder engagement"[tiab:~3] OR "Stakeholder involvement"[tiab:~3] OR "Patient and public engagement"[tiab:~3] OR Co-design[tiab] OR Co-creation[tiab] OR Co-production[tiab] OR "Citizen engagement"[tiab:~3] OR "intervention design"[tiab:~3] OR "Participatory research"[tiab:~3] OR "action research"[tiab] OR "Experienced-based co-design"[tiab:~3] OR Codesign[tiab] OR Cocreation[tiab] OR Coproduction[tiab] OR "Collaborative research"[tiab:~3] OR “citizen science”[tiab] OR "patient participation"[tiab:~3] OR "public engagement"[tiab:~3] OR "patient driven"[tiab:~3] OR "community engagement"[tiab:~3] OR “intervention development”[tiab:~3] OR "community research"[tiab:~3]

AND

exercise[Mesh] OR "motor activity"[Mesh] OR "sedentary behavior"[Mesh] OR "physical activit*"[tiab] OR "physical function*"[tiab] OR mobility[tiab] OR "exercis*"[tiab] OR "motor activit*"[tiab] OR ambulation[tiab] OR "inactiv*"[tiab] OR sedentary[tiab]

**Scopus**
21-03-2024: 1519 results
06-03-2025: 1680 results

TITLE-ABS-KEY (Stroke OR poststroke OR CVA OR {cerebrovascular accident})

AND

TITLE-ABS-KEY ( ( ( citizen* OR patient* OR stakeholder* OR public* OR communit* OR collaborat* OR intervention* OR action ) W/1 ( science OR research OR participat* OR involvement* OR engag* OR partnership* OR development* OR driven ) ) OR ( co PRE/0 ( design* OR creat* OR production* ) ) )

AND

TITLE-ABS-KEY ( exercise OR inactiv* OR mobility OR walking OR ambulation OR sedentary OR ( ( physical ) W/1 ( function* OR activ*) ) )

**Embase**
21-03-2024: 1643 results
06-03-2025: 1753 results

(‘cerebrovascular accident’/exp OR ‘poststroke’:ti,ab,kw OR ‘CVA’:ti,ab,kw OR ‘stroke’:ti,ab,kw)

AND

'citizen science'/exp OR 'participatory research'/exp OR 'patient participation'/exp OR 'stakeholder participation'/exp OR 'community participation'/exp OR (((citizen* OR patient* OR stakeholder* OR public* OR communit* OR collaborat* OR intervention* OR action) NEAR/1 (science OR research OR participat* OR involvement* OR engag* OR partnership* OR development* OR driven)):ti,ab,kw) OR ((co NEXT/1 (design OR creation OR production)):ti,ab,kw)

AND

(‘physical activity’/exp OR ‘physical inactivity’/exp OR ‘motor activity’/exp OR ‘exercise’/exp OR ‘sedentary lifestyle’/exp OR (physical NEXT/1 (activit* OR function*)):ti,ab,kw OR ‘mobility’:ti,ab,kw OR ‘exercis*’:ti,ab,kw OR ‘motor activit*’:ti,ab,kw OR ‘walking’:ti,ab,kw OR ‘ambulation’:ti,ab,kw OR ‘sedentary’:ti,ab,kw OR ‘inactiv*’:ti,ab,kw)

**Cinahl**
21-03-2024: 523 results
06-03-2025: 561 results

(MH "Stroke+") OR TI ( (poststroke OR CVA OR "cerebrovascular accident*" OR stroke) ) OR AB ( (poststroke OR CVA OR "cerebrovascular accident*" OR stroke) )

AND

( (MH "Citizen Science") OR (MH "Patient Participation+") OR (MH "Stakeholder Participation") OR (MH "Action Research") ) OR ( ( citizen* OR patient* OR stakeholder* OR public* OR communit* OR collaborat* OR intervention* OR action ) N1 ( science OR research OR participat* OR involvement* OR engag* OR partnership* OR development* OR driven ) ) OR ((co) N0 (design OR creation OR production))

AND

( (MH "Physical Activity") OR (MH "Exercise+") OR (MH "Life Style, Sedentary+") OR (MH "Motor Activity+") ) OR TI ( "physical activit*" OR "physical function*" OR mobility OR "exercis*" OR "motor activit*" OR walking OR ambulation OR sedentary OR inactiv* ) OR AB ( "physical activit*" OR "physical function*" OR mobility OR "exercis*" OR "motor activit*" OR walking OR ambulation OR sedentary OR inactiv* )

**PsycINFO**
21-03-2024: 281 results
06-03-2025: 299 results

(MH "Stroke+") OR TI ( (poststroke OR CVA OR "cerebrovascular accident*" OR stroke) ) OR AB ( (poststroke OR CVA OR "cerebrovascular accident*" OR stroke) )

AND

( (MH "Citizen Science") OR (MH "Patient Participation+") OR (MH "Stakeholder Participation") OR (MH "Action Research") ) OR ( ( citizen* OR patient* OR stakeholder* OR public* OR communit* OR collaborat* OR intervention* OR action ) N1 ( science OR research OR participat* OR involvement* OR engag* OR partnership* OR development* OR driven ) ) OR ((co) N0 (design OR creation OR production))

AND

( (MH "Physical Activity") OR (MH "Exercise+") OR (MH "Life Style, Sedentary+") OR (MH "Motor Activity+") ) OR TI ( "physical activit*" OR "physical function*" OR mobility OR "exercis*" OR "motor activit*" OR walking OR ambulation OR sedentary OR inactiv* ) OR AB ( "physical activit*" OR "physical function*" OR mobility OR "exercis*" OR "motor activit*" OR walking OR ambulation OR sedentary OR inactiv* )
