# Supplementary material for: Citizen science approaches in the development of post-stroke physical activity interventions: A scoping review
Source: PLoS One. 2025 Aug 20;20(8):e0329948. doi: 10.1371/journal.pone.0329948 (PMC12367154; doi:10.1371/journal.pone.0329948)
Supplement: S3 File — (DOCX) [file pone.0329948.s003.docx]

| **Field** | **Note** |
| --- | --- |
| Author | Names of authors |
| Year | Year published |
| Title | Publication title |
| Journal | Journal name |
| Country in which the study was conducted | If multiple countries for data collection then list all here |
| Project name | Name of project if specified |
| Brief description | Research questions, study aims and objectives |
| Participants | Specify number, age, sex |
| Short description of participants | Relevant characteristics of participants (e.g. who are the citizen scientist?: stroke patients, caregivers etc.) |
| Severity of stroke | Description of e.g. the severity of the stroke, cognitive impairment etc. |
| Inclusion/exclusion criteria | Eligibility criteria |
| Recruitment | Recruitment methods for engaging citizen scientists |
| Study design | E.g. RCT, mixed-methods, qualitative |
| Setting | What is the setting of the study/intervention (e.g. community, rehabilitation, hospital) |
| Start date/end date study | Date |
| Terminology | Which term is used to describe the citizen science approach? |
| Focus of intervention | E.g. physical activity and/or sedentary behaviour |
| Outcome measures | How is PA and/or SB measured. Specify tools used |
| Engagement of other stakeholders | Describe any policy or practice stakeholder engagement or collaboration |
| What activities are the citizen scientist involved in? | Key activities citizen scientist conducted (e.g. data collection, analysis, advocacy, dissemination, training etc.) |
| Methods of collaboration | Which methods have been used to collaborate with citizens? |
| Findings | Key findings of the study |
| Evaluation aims | Aims and objectives of evaluation, if specified |
| Evaluation methods | Evaluation methods, if specified |
| Evaluation findings | Evaluation findings, if reported including motivations to engage, experiences of participating, feasibility or utility of citizen science approaches, and key limitations or challenges of projects, where related to the citizen science approach taken |
| Impact on citizens | Reported impacts of projects upon citizen scientists, if specified (e.g. intervention adherence, patient's enjoyment, scientific literacy, health literacy, social skills, empowerment, research skills) |
