# Supplementary material for: Citizen science approaches in the development of post-stroke physical activity interventions: A scoping review
Source: PLoS One. 2025 Aug 20;20(8):e0329948. doi: 10.1371/journal.pone.0329948 (PMC12367154; doi:10.1371/journal.pone.0329948)
Supplement: S4 File — (DOCX) [file pone.0329948.s004.docx]

| **Stages of research process** | | **Non-involvement** | **Listener** | **Co-thinker** | **Advisor** | **Partner** | **Decision-maker** |
| --- | --- | --- | --- | --- | --- | --- | --- |
| **Preparation** | **Research question** |  | **Bodilsen 2023**  **Driver 2020**  **Ezeugwu & Manns 2020**  **Hall 2020**  **Heron 2021**  **Irvine 2023**  **Levy 2022**  **Lund 2012**  **Moore 2022**  **Olafsdottir 2020**  **Sakakibara 2017**  **Kwah 2024** |  | **Morris 2022** | **Ramage 2022**  **Ramage 2022** | **Bodilsen 2023**  **Driver 2020**  **Ezeugwu & Manns 2020**  **Hall 2020**  **Heron 2021**  **Irvine 2023**  **Lund 2012**  **Moore 2022**  **Morris 2022**  **Olafsdottir 2020**  **Sakakibara 2017**  **Kwah 2024** |
|  | **Study design** |  | **Bodilsen 2023**  **Driver 2020**  **Ezeugwu & Manns 2020**  **Hall 2020**  **Heron 2021**  **Irvine 2023**  **Levy 2022**  **Lund 2012**  **Moore 2022**  **Olafsdottir 2020**  **Sakakibara 2017**  **Kwah 2024** |  | **Morris 2022** | **Ramage 2022**  **Ramage 2022** | **Bodilsen 2023**  **Driver 2020**  **Ezeugwu & Manns 2020**  **Hall 2020**  **Heron 2021**  **Irvine 2023**  **Lund 2012**  **Moore 2022**  **Morris 2022**  **Olafsdottir 2020**  **Sakakibara 2017**  **Kwah 2024** |
| **Execution** | **Data collection** |  |  | **Ezeugwu & Manns 2020**  **Levy 2022**  **Sakakibara 2017** | **Driver 2020**  **Heron 2021**  **Lund 2012**  **Olafsdottir 2020** | **Bodilsen 2023**  **Bodilsen 2023**  **Hall 2020**  **Hall 2020**  **Irvine 2023**  **Irvine 2023**  **Moore 2022**  **Morris 2022**  **Morris 2022**  **Ramage 2022**  **Ramage 2022**  **Kwah 2024**  **Kwah 2024** | **Driver 2020**  **Ezeugwu & Manns 2020**  **Heron 2021**  **Lund 2012**  **Moore 2022**  **Sakakibara 2017** |
|  | **Data analysis** | **Ezeugwu & Manns 2020**  **Kwah 2024** | **Bodilsen 2023**  **Driver 2020**  **Heron 2021**  **Levy 2022**  **Olafsdottir 2020**  **Sakakibara 2017** | **Hall 2020**  **Lund 2012**  **Moore 2022**  **Morris 2022** |  | **Irvine 2023**  **Ramage 2022**  **Ramage 2022** | **Bodilsen 2023**  **Driver 2020**  **Ezeugwu & Manns 2020**  **Hall 2020**  **Heron 2021**  **Irvine 2023**  **Lund 2012**  **Moore 2022**  **Morris 2022**  **Sakakibara 2017**  **Kwah 2024** |
| **Implementation** | **Writing** | **Bodilsen 2023**  **Driver 2020**  **Ezeugwu & Manns 2020**  **Hall 2020**  **Heron 2021**  **Irvine 2023**  **Levy 2022**  **Lund 2012**  **Moore 2022**  **Morris 2022**  **Olafsdottir 2020**  **Sakakibara 2017**  **Kwah 2024** |  |  |  | **Ramage 2022**  **Ramage 2022** | **Bodilsen 2023**  **Driver 2020**  **Ezeugwu & Manns 2020**  **Hall 2020**  **Heron 2021**  **Irvine 2023**  **Lund 2012**  **Moore 2022**  **Morris 2022**  **Olafsdottir 2020**  **Sakakibara 2017**  **Kwah 2024** |
|  | **Dissemination** | **Bodilsen 2023**  **Calder 2022**  **Driver 2020**  **Ezeugwu & Manns 2020**  **Hall 2020**  **Heron 2021**  **Irvine 2023**  **Levy 2022**  **Lund 2012**  **Moore 2022**  **Morris 2022**  **Olafsdottir 2020**  **Sakakibara 2017**  **Kwah 2024** |  |  |  | **Ramage 2022**  **Ramage 2022** | **Bodilsen 2023**  **Calder 2022**  **Driver 2020**  **Ezeugwu & Manns 2020**  **Hall 2020**  **Heron 2021**  **Irvine 2023**  **Lund 2012**  **Moore 2022**  **Morris 2022**  **Olafsdottir 2020**  **Sakakibara 2017**  **Kwah 2024** |

Patient role in black, researcher role in blue
